# Polyphenols as potential metabolism mechanisms regulators in liver protection and liver cancer prevention

**DOI:** 10.1111/cpr.13346

**Published:** 2022-10-13

**Authors:** Shuangfeng Li, Shuangshuang Yin, Hui Ding, Yingying Shao, Shiyue Zhou, Weiling Pu, Lifeng Han, Tao Wang, Haiyang Yu

**Affiliations:** ^1^ State Key Laboratory of Component‐based Chinese Medicine Tianjin University of Traditional Chinese Medicine Tianjin China; ^2^ Key Laboratory of Pharmacology of Traditional Chinese Medical Formulae, Ministry of Education Tianjin University of Traditional Chinese Medicine Tianjin China; ^3^ Haihe Laboratory of Modern Chinese Medicine Tianjin China

## Abstract

**Background:**

Liver cancer is one of the common malignancies. The dysregulation of metabolism is a driver of accelerated tumourigenesis. Metabolic changes are well documented to maintain tumour growth, proliferation and survival.

Recently, a variety of polyphenols have been shown to have a crucial role both in liver disease prevention and metabolism regulation.

**Methods:**

We conducted a literature search and combined recent data with systematic analysis to comprehensively describe the molecular mechanisms that link polyphenols to metabolic regulation and their contribution in liver protection and liver cancer prevention.

**Results:**

Targeting metabolic dysregulation in organisms prevents and resists the development of liver cancer, which has important implications for identifying new therapeutic strategies for the management and treatment of cancer. Polyphenols are a class of complex compounds composed of multiple phenolic hydroxyl groups and are the main active ingredients of many natural plants. They mediate a broad spectrum of biological and pharmacological functions containing complex lipid metabolism, glucose metabolism, iron metabolism, intestinal flora imbalance, as well as the direct interaction of their metabolites with key cell‐signalling proteins. A large number of studies have found that polyphenols affect the metabolism of organisms by interfering with a variety of intracellular signals, thereby protecting the liver and reducing the risk of liver cancer.

**Conclusion:**

This review systematically illustrates that various polyphenols, including resveratrol, chlorogenic acid, caffeic acid, dihydromyricetin, quercetin, catechins, curcumin, etc., improve metabolic disorders through direct or indirect pathways to protect the liver and fight liver cancer.

## Graphical abstract

Cancer treatment has increasingly relied on drugs of natural origin. Polyphenols are a general term for polyhydroxy compounds synthesized from gallic acid or its polymers of glucitol, flavanols and their derivatives, as well as by mixing the two through the shikimic acid and malonic acid pathways. They are powerful emerging compounds for the prevention and treatment of liver cancer. Most patients with unexplained liver cancer have metabolic syndrome, therefore, regulating metabolism and preventing liver diseases such as NAFLD is one of the effective strategies to prevent liver cancer. Targeting organism metabolism is one of the pathophysiological mechanisms of anticancer drug discovery. This research analyzes that polyphenols improve metabolic disorders through direct or indirect pathways to protect the liver and fight liver cancer.

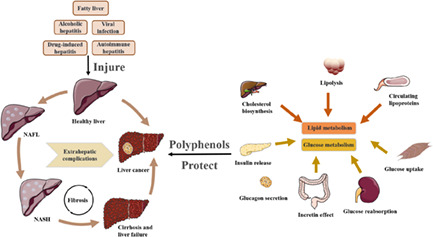

## 1 INTRODUCTION

**FIGURE 1:**
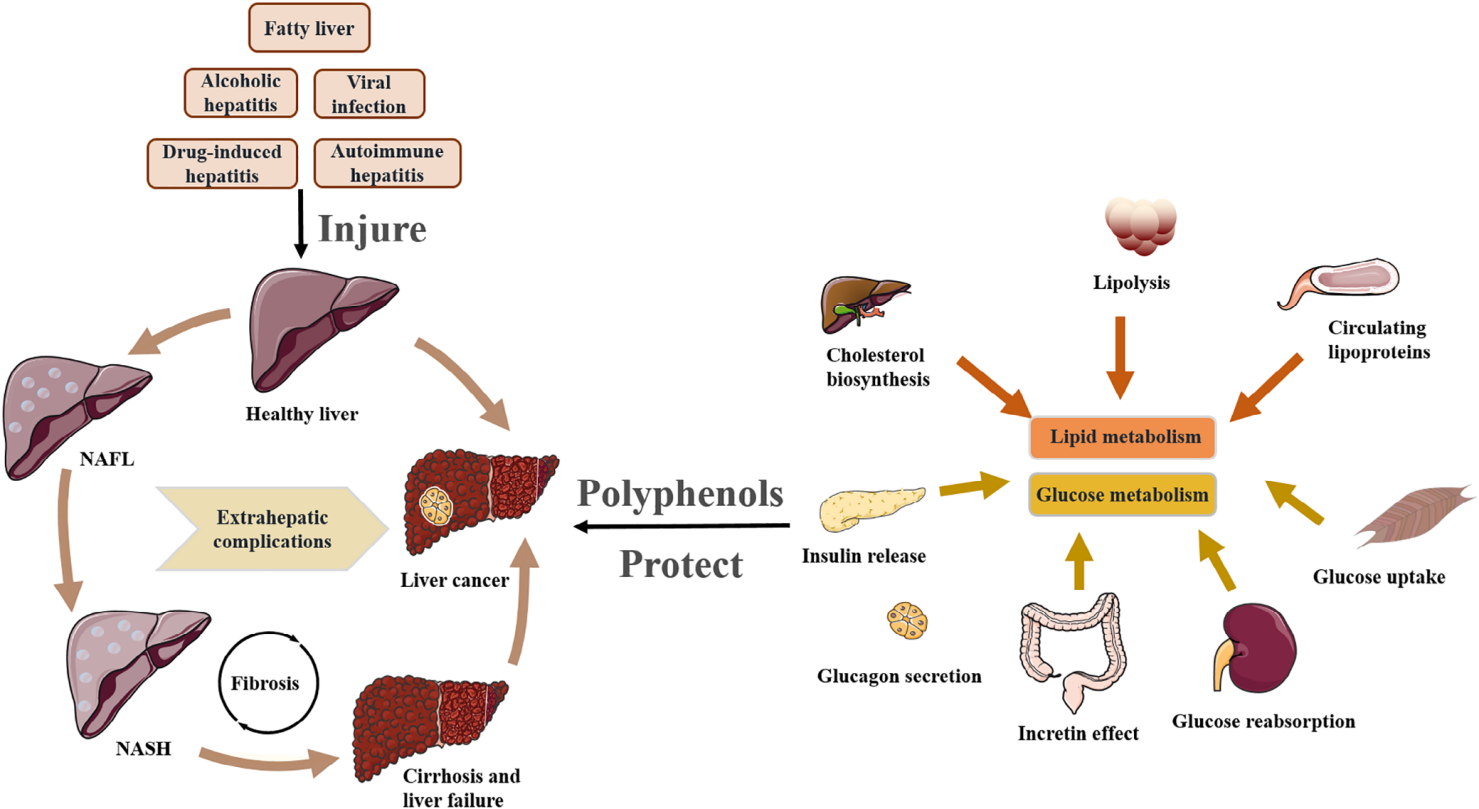
The development process of liver cancer, and polyphenols act on lipid and glucose metabolism. A variety of risk factors cause liver diseases, leading to the deterioration of NAFLD to NASH, and even to liver cancer which is also affected by the extrahepatic complications (left). Polyphenols act on functional organs related to lipid and glucose metabolism, thereby protecting the liver and liver cancer (right).

Global cancer morbidity and mortality are expeditiously increasing.^1^, ^2^ Over the past decade, liver cancer has become one of the most common malignant tumours in the world.^3^, ^4^ In the latest global cancer statistics, liver cancer ranks third in mortality, accounting for 8.3% of the total global cancer deaths and is the second leading cause of cancer deaths among males worldwide.^5^ Viral hepatitis, liver cirrhosis, aflatoxin and other chemical preparations, as well as geographically related water or soil factors are all important elements leading to hepatocarcinoma.^6^, ^7^, ^8^ In most patients, hepatocarcinoma appears with cirrhosis and is attributed to multiple risk factors, including infections, usually infected with hepatitis B or C virus, excessive alcohol consumption and non‐alcoholic fatty liver disease (NAFLD).^3^, ^9^, ^10^ There are many kinds of liver diseases, such as liver cancer, fatty liver, liver cirrhosis, alcoholic liver disease, NAFLD, hepatitis, liver failure, chronic liver disease, etc.^11^, ^12^ Alcohol consumption and infection are two common factors of liver disease. NAFLD is a metabolic syndrome characterized by excessive fat deposition in hepatocytes and is a metabolic stress‐induced liver injury closely related to insulin resistance and genetic susceptibility. Most patients with unexplained liver cancer have metabolic syndrome, therefore, NAFLD has gradually become one of the most important causes of liver cancer development.^13^, ^14^ The initial stage of NAFLD is the nonalcoholic fatty liver (NAFL), and in the middle stage, it will evolve into nonalcoholic steatohepatitis (NASH), and a small number of patients will progress to liver fibrosis, liver cirrhosis and even hepatocellular carcinoma in the later stage.^15^ NASH is a severe NAFLD, a liver complication caused by inflammation and liver cell damage. Unlike simple fatty liver, NASH leads to complications such as liver cancer, cirrhosis and liver damage (Figure 1, left). The pathogenesis of NAFLD‐related hepatocellular carcinoma is closely related to metabolism.^16^, ^17^

The valid treatment of hepatocarcinoma depends on early diagnosis and appropriate monitoring of patient response to therapies, also noticing pathways and mechanisms regulated during tumorigenesis.^18^, ^19^, ^20^ The body's glucose metabolism, lipid metabolism and intestinal flora imbalance are intimately related to liver cancer. In approximately two‐thirds of liver cancers, significant changes in lipid metabolism have been described, mainly in large cellular lipid droplets rich in neutral lipids. In particular, diffuse fat alterations may occur in early HCC as compared to others that typically do not show any visible lipid accumulation.^21^ Glucose metabolic disorders are one of the hallmarks of expeditious cell proliferation in tumour cells. Proliferative tumour cells are altered by metabolizing carbohydrates, lipids, and peptides to accommodate growing energy needs, and perform protein and nucleic acid biosynthesis and membrane biogenesis.^22^ Liver cancer cells exhibit an unwonted metabolic phenotype, and tumour development is extraordinarily dependent on glycolysis. Even in the case of sufficient oxygen, the tumour will undergo a contraction in oxidative phosphorylation, which is a metabolic reprogramming process known as aerobic glycolysis (‘Warburg effect’).^23^, ^24^, ^25^ Studies have demonstrated that the hepatoprotective and anti‐inflammatory benefits of catechins‐enriched green tea extract (GTE) prevent hepatocellular carcinoma (HCC) progression by inhibiting NASH‐related liver damage and promoting oncogenic responses. GTE reduces diethylnitrosamine (DEN) induced hepatic lipid accumulation, lipid peroxidation and liver injury (Figure 1, right).^26^

Nowadays, the treatment of cancer is progressively dependent on drugs from natural sources, and natural drugs have been accepted as effective sources for developing anti‐cancer and neoadjuvant drugs.^27^, ^28^, ^29^ Polyphenols, a class of complex secondary metabolites of phenols that cannot be synthesized by human bodies and need to be obtained through diet, are a general term for substances with a combination of benzene rings and various hydroxyl structures.^30^, ^31^ Polyphenols possess multiple biological functions, including anti‐cancer, liver protection, anti‐oxidant, anti‐allergic, anti‐inflammatory, anti‐mutagenic and anti‐proliferative effects.^32^ There is evidence that polyphenols inhibit the development of liver cancer and protect the liver by altering metabolism.^30^, ^33^ However, there is a lack of systematic comprehensive work to specifically integrate the regulation of polyphenols on liver cancer and liver protection through metabolism. Therefore, this review focuses on an all‐inclusive and in‐depth understanding of how natural polyphenols protect the liver and restrain hepatocarcinoma development by affecting metabolism.

## 2 POLYPHENOLS PROTECT THE LIVER AND AGAINST HEPATOCARCINOMA

**FIGURE 2:**
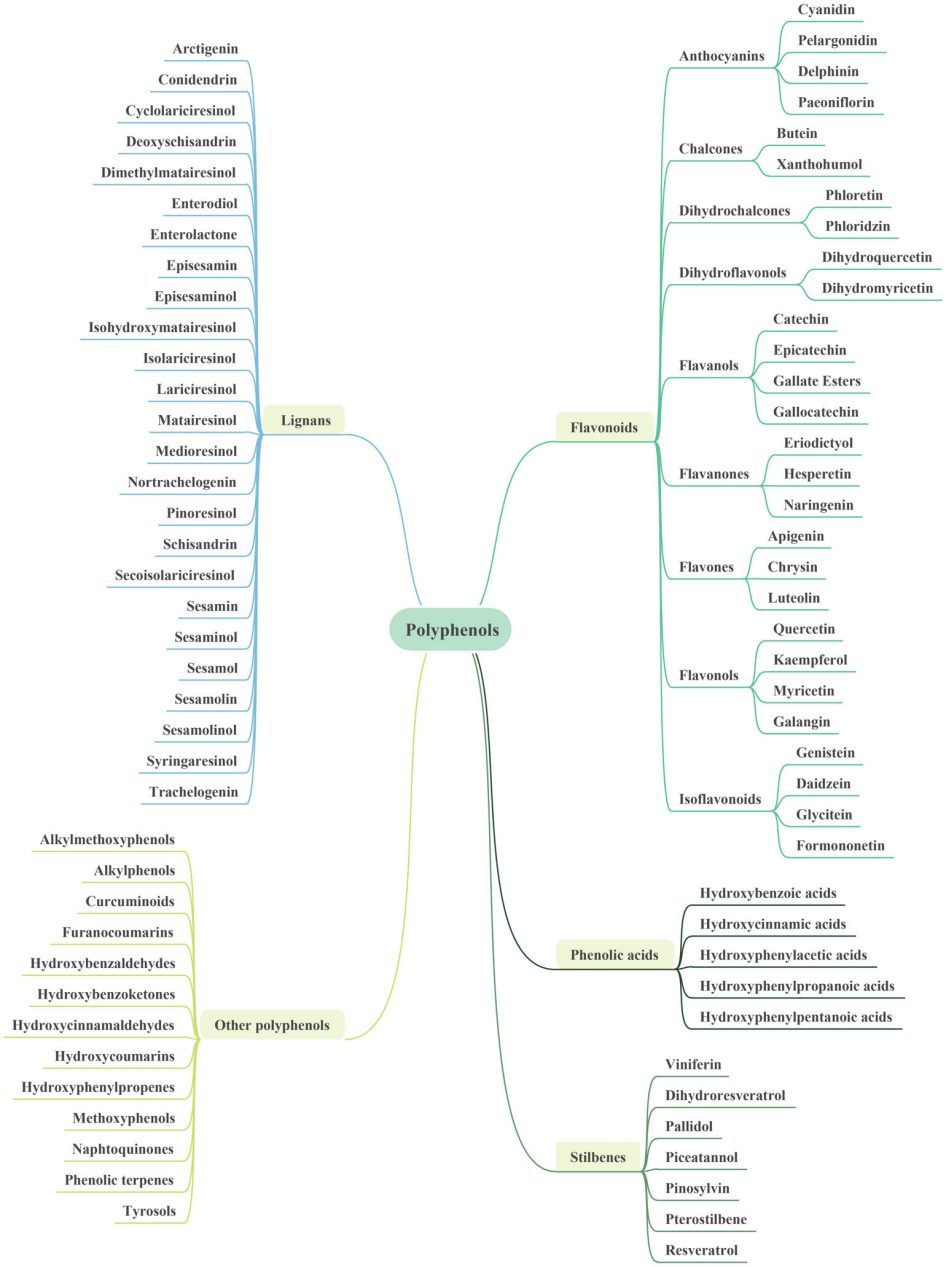
The classification of polyphenolic compounds. There are abundant kinds of polyphenols in nature, thus their classification is complex, mainly including Flavonoids, Phenolic acids, Stilbenes, Lignans and other polyphenols.

Polyphenols are characterized by low toxicity and broad‐spectrum pharmacological activities such as anti‐oxidation, anti‐atherosclerosis, anti‐hypertension, hypoglycemic, anti‐inflammatory, anti‐bacterial, anti‐virus, anti‐cancer, etc., therefore are widely used in medical treatment, new drug development, food and health care.^34^, ^35^ At present, more than 8000 polyphenols have been found in nature, and the most common sources of phenolic compounds can be found in many daily ingestible foods such as fruits, vegetables, tea, coffee, cocoa, beans and grains.^36^ With regard to chemical structure, their basic carbon structure is composed of 2‐phenylbenzopyran and polyhydroxyl, which is characterized by the polyhydroxy substituent of the benzene ring.^37^ The complex structure of polyphenols is embodied in that some can be combined with monosaccharides or polysaccharides to form glycosides, and some exist in the form of derivatives. Multiple ortho‐phenolic hydroxyl groups of polyphenols have complexation reactions with metal ions, which bind to proteins through hydrophobic bonds and multi‐site hydrogen bonds, and undergo molecular complex reactions with other biological macromolecules, such as alkaloid and polysaccharides.^38^ Polyphenol compounds are divided into four major categories according to their structural formulas and chemical groups, (1) Flavonoids, including flavonols, isoflavones, chalcones, anthocyanins, catechins, etc.; (2) Phenolic acids, including hydroxybenzoic acid, hydroxyphenylacetic acid, hydroxycinnamic acid, etc.; (3) Lignans, including diepoxy lignans, monoepoxy lignans, cyclolignans, new lignans, etc.; (4) Stilbenes, including trans‐stilbene, trans‐resveratrol, etc.^39^, ^40^ The detailed classification and types of polyphenols are shown in Figure 2.

Plenteous literature has reported that a variety of polyphenols possess liver‐protection and anti‐hepatocarcinoma activities, and their mechanisms are also diverse, for example, affecting the expression of some proteins or genes to stymie the proliferation, autophagy, apoptosis, metastasis, metabolic cycle, angiogenesis and senescence of tumour cells.^41^, ^42^ Mulberry fruits, containing polyphenols to scavenge free radicals and slash inflammation, prevent liver damage caused by alcohol or CCl_4_. It was observed that the mulberry polyphenol extract lessened DEN‐induced serum aspartate aminotransferase (AST) and alanine aminotransferase (ALT), cleaved caspase, HCC marker, Ser‐15‐p53 and Ser‐46‐p53. P53‐dependent apoptosis and p53‐inoperative autophagy cause the death of HCC cells and inhibit cell growth of HepG2 cells and Hep3B cells.^43^ Moreover, oleocanthal and aglycone which are polyphenols derived from olive oil interfere with the proliferation and death of HepG2, Huh7 and Hep3B. This effect is related to the regulation of autophagy and can be enhanced by tumour necrosis factor‐alpha (TNFα).^44^ It was reported that apigenin (APG) brought into anti‐proliferative effect of HCC, related to apoptosis. APG induced apoptosis‐related vimentin will become a key factor in the mechanism of HCC and Huh7 cells through anti‐angiogenesis and anti‐migration. APG down‐regulates the expression level of collagen type I, which cooperates with vimentin to promote cell migration and reduces the release of vascular endothelial growth factor (VEGF) and matrix metalloproteinase‐8 (MMP‐8) closely related to the angiogenic activity.^45^ Cumulatively, plant‐derived polyphenols and their synthetic or semi‐synthetic derivatives have pleiotropic biological activities. Resveratrol, quercetin, catechins, curcumin and serotonin influence mitochondrial function by regulating apoptosis, the activity of electron transporter proteins and mitochondrial membrane potential.^46^ Consequently, the mechanism by which polyphenols induce cancer cell death directly or indirectly involves the regulation of mitochondrial function and energy metabolism. These findings clearly indicate that the potential value of polyphenols as chemotherapeutic agents is to affect the metabolic processes of organisms.

### 2.1 Phenolic acids

Phenolic acids are one of the central categories of plant polyphenolic compounds, referring to aromatic carboxylic acid compounds substituted with multiple phenolic hydroxyl groups on one benzene ring.^47^, ^48^ Phenolic acids exist in complex forms, mainly combined with ester linkage, glucoside linkage, ether linkage and other substances to form assorted esters of saccharides and organic acids, while rarely exist in free form. Phenolic acids can be divided into two subgroups, hydrobenzoic and hydrocinnamic acids, according to their carbon skeleton structure. The five prevailing hydroxybenzoic acids are p‐hydroxybenzoic acid, protocatechuic acid, vanillic acid, syringic acid and gallic acid. Moreover, the most common hydroxycinnamic acids include caffeic acid, ferulic acid, p‐coumaric acid and erucic acid. In addition, a few phenolic acids such as chlorogenic acid, ellagic acid, salvianolic acid and ginkgo phenolic acid do not belong to any of these two categories.^47^, ^48^, ^49^ Multifarious phenolic acids have been found to have health protection effects, such as anti‐tumour, anti‐oxidation, anti‐inflammatory, anti‐viral, anti‐mutagenic, bacteriostatic activity, liver and cardiovascular protection, etc.^50^ Pharmacological experiments have proved that they have vigorous anti‐cancer abilities both in vitro and in vivo. The fundamental structural motifs required for anti‐cancer activity include aromatic rings, unsaturated substituted chains, and the number and position of free hydroxyl groups.^51^ Acid substances are often extracted from plant seeds, for instance, chicory seed extracts contain phenolic acids such as caffeic acid, chlorogenic acid and chitosan acid. By up‐regulating the expression levels of peroxisome proliferators activated receptor alpha (PPARα) and sterol regulatory element binding protein‐1 (SREBP‐1), two important gene regulators involved in hepatic lipid metabolism could recuperate diabetic and oleic acid‐induced NAFLD or NASH, in turn affecting liver cancer.^52^ By the same token, caffeic acid, chlorogenic acid and cinnamic acid upgrade glucose metabolism by regulating glycogen production and gluconeogenesis, stymieing protein glycosylation and manufacturing advanced glycosylation end products.^53^ There have been many reports in the literature that phenolic acids affect metabolic regulation in organisms, thereby obstructing cancer progression.

Chlorogenic acids (CGA) refer to a family of ester compounds formed by the condensation of quinic acid (QA) and trans‐cinnamic acid (t‐CA), which routinely are caffeic acid (CA), p‐coumaric acid (p‐CoA) and ferulic acid (FA).^54^, ^55^, ^56^ CGA plays a key role in the regulation of lipid and glucose metabolism by regulating glucose‐6‐phosphatase (G‐6‐P) activity and adiponectin receptor signalling pathway, thereby conducing to treat many diseases, such as liver steatosis, diabetes and obesity.^57^

CA, also known as 3,4‐dihydroxycinnamic acid, has a hydroxybenzene acrylic structure.^58^, ^59^ CA prevents acetaminophen (APAP)‐induced hepatotoxicity by reducing Kelch‐like ECH associated protein 1 (Keap1) expression and inhibiting the binding of Keap1 to nuclear factor‐erythroid 2‐related factor 2 (Nrf2), thereby activating Nrf2 and leading to increased expression of anti‐oxidant signals such as heme oxygenase 1 (HO‐1) and NAD(P)H‐quinone oxidoreductase‐1 (NQO1).^60^, ^61^ Treating with CA attenuated liver microcirculatory disturbance and oxidative injury, inhibits sirtuin 3 (SIRT3) down‐expression and up‐regulates mitochondrial respiratory chain (MCT) activity.^62^

### 2.2 Flavonoids

Flavonoids are secondary metabolites of polyphenols, having bountiful biological properties, including anti‐oxidant, anti‐bacterial, anti‐viral, anti‐inflammatory, anti‐cancer and neuroprotective activities, broadly in fruits, vegetables and beverages derived from plants.^63^ It is recognized that flavonoids undergo extensive metabolism before entering systemic circulation. The absorbed flavonoids bind to albumin and are transported to the liver through the portal vein.^64^ It is reported that they alter cellular glucose uptake, metabolism and glucose transporter gene expression in the HepG2 cell model.^65^ A growing body of evidence shows that at least in part through the immunomodulatory, anti‐inflammatory and anti‐oxidant properties of these compounds, the intake of flavonoids and related compounds could hamper cancer and chronic diseases including cardiovascular disease, type II diabetes (TD2M) and NAFLD. Flavonols have propitious effects on lipid accumulation, inflammation, fibrosis, nitrosation/oxidative stress and insulin resistance associated with NAFLD.^66^, ^67^ Dihydromyricetin (DMY), chemically named (2R,3R)‐3,5,7‐trihydroxy‐2‐(3,4,5‐trihydroxyphenyl)‐2,3‐dihydrochromen‐4‐one, is a dominant bioactive flavonoid isolated from the leaves of the traditional Chinese medicine Ampelopsis grossedentata.^68^, ^69^ DMY can be used as a protective agent to avert liver damage, including liver damage induced by acetaminophen, ethanol and carbon tetrachloride. Numerous studies support that DMY promotes liver health by precluding lipid metabolism, boosting ethanol metabolism and preventing inflammation, thereby abridging ethanol liver damage through dietary supplements.^70^ Quercetin (QUE) is a plant‐derived flavonoid glycoside.^71^ More and more evidence shows that the naturally occurring QUE can depress blood lipids, resulting in liver protection, preventing the deterioration of cancer as well as influencing the regulation of signal transduction molecules.^72^, ^73^ Both in vitro and in vivo, QUE supplementation can ameliorate sundry outcomes related to metabolic syndrome.^74^ Green tea is an affluent source of flavonoids, containing many catechin polyphenols, of which the four main types are epicatechin (EC), epigallocatechin (EGC), epicatechin gallate (ECG) and epigallate catechin gallate (EGCG).^75^, ^76^ EC reduces hepatic oxidative damage by activating the Nrf2 anti‐oxidant pathway and suppresses hepatic inflammatory damage by abrogating the heat shock protein 60 (HSP60)‐initiated nuclear factor‐kappaB (NF‐κB) signalling pathway, thereby attenuating MCT‐induced hepatic sinusoidal obstruction syndrome (HSOS).^77^

### 2.3 Curcumin

Curcumin (CUR) is a yellow polyphenol compound derived from the plant *Curcuma longa* L., commonly known as difluoroformylmethane, extensively used as a spice and colouring agent in food. It has omnifarious pharmacological activities, including anti‐inflammatory, anti‐oxidant, anti‐tumour and lipid‐lowering activities. At the same time, CUR has been used as a natural medicine for dyspepsia, peptic ulcers, skin diseases, liver and urinary system diseases for a long time.^78^, ^79^ Recent data provide additional evidence that CUR can also be used as a drug or adjuvant for traditional chemotherapy for cancer treatment, encompassing liver cancer, lung cancer, cervical cancer, prostate cancer, breast cancer and bone cancer.^80^ In tumour cells, the pleiotropic combination of CUR can prevent many pivotal metabolic processes and activate cell death pathways through different molecular triggers, thereby jointly enhancing tumour‐killing efficacy. Collectively, CUR has a cytoprotective effect on normal cells due to its vigorous anti‐oxidant properties. It has been reported to work synergistically with various types of chemotherapeutic drugs to administer adjuvant effects.^81^ Studies have shown that CUR regulates glucose and lipid metabolism and epithelial‐to‐mesenchymal transition (EMT) based on the PI3K/AKT pathway.^82^

### 2.4 1,2‐stilbene compound—resveratrol

**FIGURE 3:**
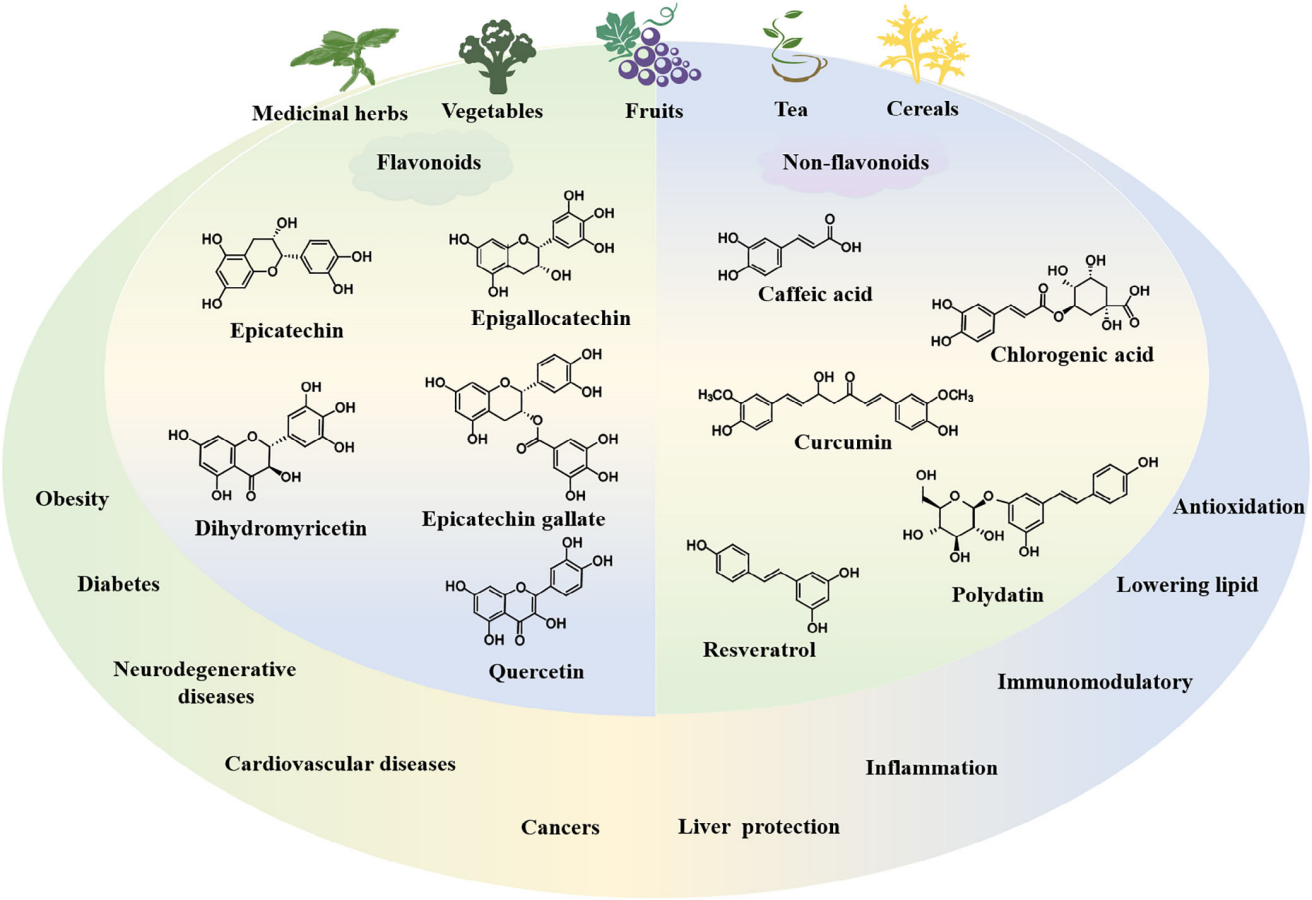
The sources, pharmacological effects and structural formulas of common polyphenolic compounds.

Resveratrol (RES), 3,5,4′‐trihydroxy‐trans‐stilbene, is a 14‐carbon backbone of stilbene. RES is a natural polyphenol compound found in many plants, such as grapes, berries and peanuts, which have been substantiated to have anti‐cancer, heart‐protecting, anti‐senescence, anti‐inflammatory and anti‐oxidant properties.^83^, ^84^ RES can dwindle liver damage caused by alcohol and hepatotoxic drugs, ameliorate lipid metabolism, debase lipid accumulation and safeguard HepG2 cells, enhance anti‐oxidant capacity and restore mitochondrial respiratory chain activity (Figure 3).^85^

## 3 SPECIFIC MECHANISMS OF POLYPHENOLS AFFECTING METABOLISM

### 3.1 Direct regulation of lipid metabolism

Lipids are vital nutrients for the human body, comprising fats, lipids and their derivatives. Lipid metabolism is not only associated with cell energy supply, but also intently associated with cell signal transduction in maintaining cell membrane structure. Reprogramming lipid metabolism is essential for cell growth and carcinogenesis.^86^, ^87^ Therefore, the emergence of liver cancer is always accompanied by abnormal lipid metabolism, which festers cancer. There is a set of finely regulated lipid metabolism networks in the body to coordinate the lipid metabolism process in order to maintain the normal structure and function of cells, clearly demonstrating the fundamentality of metabolic changes in transforming cells from the early stages of cancer development.^88^ Tumour cells often exhibit high lipid uptake rates and de novo lipogenesis. The plasma levels of triglycerides, free fatty acids, cholesterol, high/low density lipoprotein and apolipoprotein are dramatically abridged in most patients with hepatocarcinoma. Due to the rapid proliferation of tumour cells, in order to ensure the energy supply and lipid synthesis, desaturation and transport will be increased in tumour tissues, and the proliferation, death, invasion, migration and angiogenesis of tumours will be affected through the relevant pathways.^89^

Currently, the researches on the mechanism of polyphenols regulating lipid metabolism focus on fundamental enzyme activities and related gene expression during adipocyte differentiation, lipid synthesis, decomposition and excretion.^90^ Adenosine 5′‐monophosphate‐activated protein kinase (AMPK) also plays a pushing role in preventing liver cancer and regulating liver cancer‐related genes and pathways, and dietary polyphenols rectify lipid metabolism through AMPK‐targeted molecules.^91^ It is indicated that the molecular model for the role of DMY in paring metabolic disorders and NASH first episode is by the activation of AMPK and the regulatory contribution to downstream proteins involved in the formation of new fat and lipid oxidation. AMPK stymies the expression of SREBP‐1c through phosphorylation, which reduces the activity of mechanistic target of rapamycin (mTOR) complex or liver X receptors (LXRs), then dwindles the expression of fatty acid synthase (FAS) and subsequently impedes lipid synthesis. Other studies have confirmed that, with DMY preconditioning, the PPARs and SREBP‐1C signalling pathway significantly ameliorates lipid dysregulation, which manifests in decreased fatty acid oxidation and leads to lipid accumulation in the liver.^92^, ^93^ RES is a potent SIRT1 agonist, while AMPK has been shown to be closely associated with insulin resistance and hepatic steatosis. This effect may be related to the activated AMPK‐Lipin1 signalling, the enhanced phosphorylation of AMPK and acetyl‐CoA carboxylation (ACC), as well as the down‐regulation of the expression of SREBP‐1C and Lipin1, thereby obstructing adipogenesis and escalating energy metabolism.^94^, ^95^ RES weakens hepatic steatosis and lipid metabolic disorder in KKAy mice, possibly by up‐regulating SIRT1 expression and the phosphorylation of AMPK.^96^ Resveratrol glucuronic acid (GRES), one of the major metabolites of RES in the liver, also helps to reduce the total cholesterol content.^97^ Due to its advantageous effects on substance metabolism and energy metabolism, RES is considered as a promising new therapy for metabolic diseases. Specifically, CGA can accelerate the release of free fatty acids (FFA) from hepatocytes via mobilizing the hepatic excretion promoted by PPARα, preventing hepatic steatosis, and can reinforce exogenous triglyceride (TG) hydrolysis rate in the liver by stimulating the hepatic lipase.^98^, ^99^ Tea polyphenols are extracted from tea leaves. The fact that they possess lipid‐lowering properties is mainly because they contain a large amount of catechins, which can abate the solubility and the absorption of cholesterol in the body.^100^ EGCG and ECG can ascent the expression of low density lipoprotein (LDL) receptor protein and active SREBP‐2 in HepG2 cells, and subsequently provoke the transcription of LDL receptor genes, which may explain the cholesterol‐lowering effect of green tea.^101^ Besides, EGCG treatment altered the expression of many genes embroiled in cholesterol metabolism in human liver cancer cells. The strongest effect of EGCG is to up‐regulate LDL receptors. Another study showed that EGCG significantly elevated rat bile cholesterol and phospholipid secretion, reduced liver very low density lipoprotein (VLDL) production, and inhibited the ethinyl estradiol on liver cholesterol accumulation and liver weight. These effects are attributed to the up‐regulation of cholesterol transporters ATP‐binding cassette subfamily G5 and G8 (ABCG5/8) and scavenger receptor class B type 1 (SR‐B1), as well as down‐regulation of acyl‐coenzyme A cholesterol acyltransferase‐2 (ACAT‐2).^102^ QUE advantageously alters the expression of genes related to lipid metabolism, resisting insulin resistance and inflammation, accordingly depressing lipid accumulation and serum transaminase levels in the liver. This abridged accumulation and relieved liver swelling has been reported in the liver of db/db mice by restoring serum total bile acid and decreasing liver total bile acid, with rehabilitating abnormal liver enzymes such as superoxide dismutase, catalase and glutathione. At the same time, QUE therapy activates the farnesoid X receptor 1/Takeda G‐protein‐coupled receptor 5 signalling pathway, which improves TD2M‐induced NAFLD through anti‐oxidant, anti‐inflammatory and lipid metabolism.^103^ The specific and direct binding of polyphenols to miRNAs emerges as a novel post‐transcriptional mechanism through which polyphenols can regulate metabolism. RES and EGCG unswervingly bind miR‐33a and miR‐122, exerting a hypolipidemic effect by down‐regulating miR‐33a and miR‐122, correspondingly increasing the miR‐33a target gene ATP‐binding cassette transporter A1 (ABCA1) and inhibiting the miR‐122 indirect target gene FAS in the liver.^104^

### 3.2 Direct regulation of glucose metabolism

Glucose metabolism is the prime source of cell energy, and it is essential to retain normal glucose balance and dynamic balance. Glucose metabolism mainly refers to a series of complex chemical reactions of glucose in the body, whose catabolic mode is predominantly affected by the oxygen supply status.^105^ Glucose metabolism disorderliness associated with many diseases such as diabetes and cancer, which means that metabolic reprogramming from oxidative phosphorylation to glycolysis plays an imperative role in the development of cancer.

**FIGURE 4:**
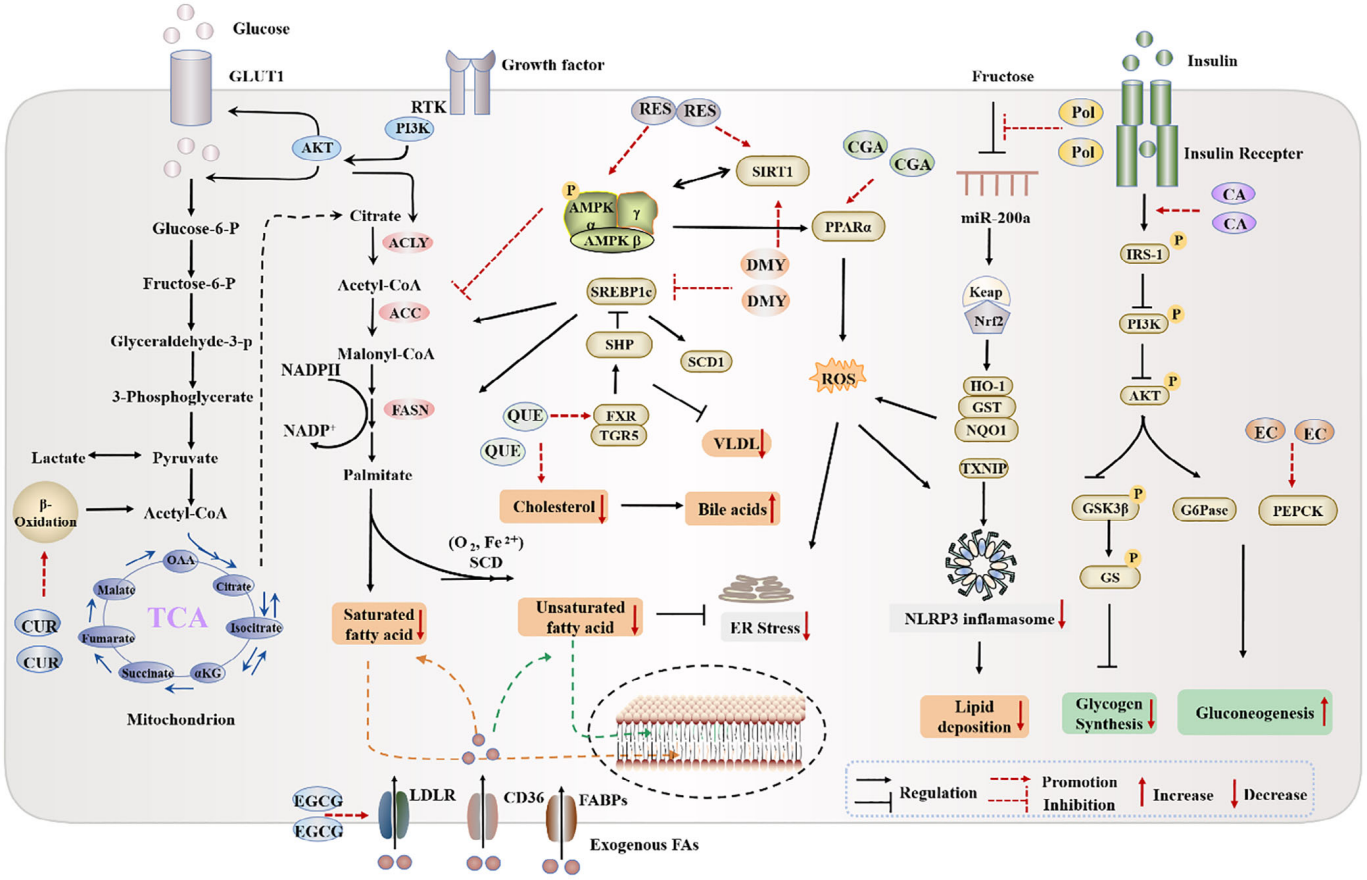
The schematic illustration of polyphenols inhibiting liver cancer development by regulating lipid and glucose metabolism. The figure summarizes the main mechanisms involved in polyphenol‐induced glycolipid metabolism in CRC. It can be seen that polyphenols mainly affect fatty acid metabolism, cholesterol metabolism, glucose metabolism, insulin resistance, etc., and show great potential in glucose and lipid metabolism.

Polyphenols, a promising regulator of glucose homeostasis, participate in the body's glucose absorption and metabolism in a variety of ways. A growing quantity of research has suggested that polyphenols can adjust enzyme activity, improve insulin sensitivity, stimulate insulin secretion, strengthen glucose uptake and intestinal hormones secretion in tissues to influence glucose metabolism, in turn, affecting the development of liver cancer.^106^, ^107^ CA activates insulin signalling paths through AKT to promote glucose intake and glycogen synthesis in the liver, as well as induces phosphorylation of PI3K, insulin receptor substrate‐1 (IRS‐1) and AMPK.^108^, ^109^ CA and its main phenolic acids can effectively stimulate glucose transport in skeletal muscles, the glucose intake and glucose transporter type 4 (GLUT4) translocation do not count on insulin‐dependent pathways, but they can revamp sensitivity in insulin treatment of HepG2 cells, and prevent or delay potential liver dysfunction by interfering insulin signalling barriers and regulating glucose consumption. CA or its methyl and ethyl esters are mediated by AMPK phosphorylation and activation to reduce glucose production and lipogenesis in liver cells. AMPK has a wide range of roles in the balance of carbohydrate and lipid homeostasis, down‐regulating the vital gluconeogenesis enzymes phosphoenolpyruvate carboxykinase (PEPCK) and G‐6‐P, restoring the cellular energy balance in the liver by activating catabolic, such as glycolysis and fatty acid oxidation, and shutting down ATP consumption, such as cholesterol synthesis, adipogenesis and gluconeogenesis.^110^, ^111^ It is reported that CA also suppressed lactic acid through monocarboxylate transporter movement, the same effect as ferulic acid. The excessive intracellular lactic acid accumulation inhibits glycolysis and cell growth by inhibiting phosphofructokinase, the rate‐limiting enzyme of glycolysis.^112^ There is evidence that EGCG restrains insulin resistance and promotes glucose uptake by enhancing GLUT4 transport in skeletal muscle cells, thereby attenuating the β‐cell release of insulin in mouse and human islet cells, and mending the insulin sensitivity of HepG2 cells by potentially lessening ROS‐induced JNK/IRS1/AKT/GSK signalling.^113^, ^114^, ^115^ In addition, EGCG subdues gluconeogenesis, glycogenolysis, mitochondrial activity and activates AMPK. It can suppress hepatic glucose production (HGP) towards hepatic glucose homeostasis by down‐regulating the protein kinase A (PKA) signalling pathway, antagonizing glucagon signalling and stifling the hepatic transcription factor forkhead box O1 (FoxO1) via Ser273.^116^ Cells pretreated with EC can antagonize the insulin signalling impediment triggered by high glucose via averting the inhibition of tyrosine phosphorylated and total insulin receptor (IR), IRS‐1 and IRS‐2 levels, the decrease of PI3K/AKT and AMPK pathways, and the increase of IRS‐1 Ser636/639 phosphorylation levels. This pretreatment also brings back the levels of GLUT‐2 to control values, and safeguards HepG2 functionality by inflecting glucose production and uptake, along with glycogen content.^117^ Increased glycolysis, one of the main sources of cellular energy supply, in tumour cells is associated with increased risk of tumour progression and death, thus, disruption of glycolysis could serve as a target for inhibiting tumour progression. QUE attenuates the proliferation of glycolysis‐addicted HCC cells by reducing the hexokinase 2 (HK2) and AKT–mTOR pathways.^118^ In all, polyphenols make a big difference in glucose metabolism (Figure 4).

### 3.3 Polyphenols affect metabolism through oxidative stress

Plant polyphenols are the most abundant natural antioxidants. It is found that the ortho‐phenolic hydroxyl groups contained in polyphenols are easily oxidized, causing a strong ability to capture free radicals such as reactive oxygen species (ROS), so that plant polyphenols exhibit decent anti‐oxidant activity. Polyphenols directly react with ROS to scavenge free radicals or integrate with metal ions, and inhibit oxidase activity to block the production of ROS, while having an indirect anti‐oxidant effect by protecting the body's endogenous anti‐oxidant enzymes.^119^, ^120^ EC, ECG and EGCG can directly influence glutathione metabolism and cytochrome P450 2E1 (CYP2E1) activity by modifying the production of subcellular ROS to prevent molecular degradation under oxidative stress.^121^ EGCG also declines hepatic and plasma lipid peroxidation products and increases glutathione levels, resulting in significant reductions in triglyceride and glucose levels.^113^, ^114^, ^115^ Furthermore, CGA can memorably attenuate the activity of FAS, 3‐hydroxy‐3‐methylglutaryl CoA reductase and acyl‐CoA: cholesterol acyltransferase activities, which can increase fatty acids beta‐oxidative activity and expression of PPARs in the liver.^122^ In addition, studies have confirmed that CUR lessens the accumulation of triglycerides in the liver of rats by up‐regulating the expression of acyl‐CoA oxidase mRNA and promoting β‐oxidation of peroxisome fatty acids. CUR inhibits the activation of nuclear factor B by blocking I‐B kinase and cyclooxygenase 2 (COX2).^123^, ^124^ One of the consequences of hepatic oxidative stress is dysregulated Ca^2+^ homeostasis, which in hepatocytes leads to irreversible cellular damage. Transient Receptor Potential Melastatin 2 (TRPM2) is activated in hepatocytes by H_2_O_2_ or paracetamol involving the formation of ADP‐ribose (ADPR), the main agonist of TRPM2, in response to oxidative stress‐induced DNA damage. As a novel inhibitor of TRPM2 channel, CUR acts as an anti‐oxidant and free radical scavenger, decreasing damage to mitochondria and DNA, thereby retarding the production of ADPR.^125^ In all, CUR not only exhibits anti‐oxidant activity, but also exhibits anti‐radical activity. Oxidative stress is a hallmark of many liver diseases, including viral and drug‐induced hepatitis, ischemia–reperfusion injury and nonalcoholic steatohepatitis.^126^ Polydatin, a polyphenol found in Polygonum cuspidatum rhizome, has anti‐oxidant, anti‐inflammatory and anti‐hyperlipidemic effects. It protects fructose‐induced liver inflammation and lipid deposition, quells Keap1 level and activates Nrf2 anti‐oxidative pathways by increasing miR‐200a expression and then blocks ROS‐driven thioredoxin interacting protein (TXNIP) to inhibit NLRP3 inflammasome activation and regulates lipid metabolism‐related proteins. These findings provide a novel pathological mechanism for fructose‐induced redox state imbalance.^127^ It can be seen that the researches on the improvement of the anti‐oxidant capacity of the body by polyphenols mainly focus on the scavenging of free radicals and the improvement of the activity of anti‐oxidant enzymes.

### 3.4 Polyphenols affect metabolism through the mitochondrial pathway

Mitochondria are the main sites of cellular energy metabolism and signal transduction, closely related to basic life activities such as cell proliferation, aging and apoptosis.^128^ Polyphenols can disrupt mitochondrial function by modulating mitochondria, disorganizing mitochondrial energy metabolism, or inhibiting the mitochondrial respiratory chain. The cinnamic acid and p‐coumaric acid of conjugated CA and its precursors to triphenylphosphine (TPP) cations protect liver mitochondria from lipid peroxidation and also help to reduce hydrogen peroxide levels and regulate anti‐oxidant enzymes. Mitochondria‐targeted HCA derivatives (MitoHCAs), the conjugated p‐coumaric, caffeic, ferulic and cinnamic acid to TPP cations, both with and without Ca2+ induce mitochondrial dysfunction by triggering mitochondrial swelling, foundering mitochondrial membrane potential and leading to cytochrome c release. Especially, mitochondrial permeability transition pore (mPTP) maybe participate in the anti‐proliferative activity of MitoHCAs, which is manifested by cyclosporin A, an inhibitor of mPTP, reducing mitochondrial damage and apoptosis.^129^ QUE elevates frataxin‐mediated phosphatase and tensin homologue (PTEN)‐induced putative kinase1 (PINK1)/Parkin (E3 ubiquitin ligase PARK2)‐dependent mitochondrial phagocytosis, and then normalizes liver lipid metabolism interfered by HFD in vivo and in vitro. The supplementation of QUE enormously reverses HFD‐induced hepatic lipid metabolism disorder and mitochondrial damage, which is manifested by the amelioration of mitochondrial morphology and function.^130^ RES in the body by increasing the number of mitochondria to adjust lipid regulating enzyme activity, leads to expressing and phosphorylating of AMPKα, and increasingly expresses acyl‐CoA synthetase, carnitine palmitoyl transferase‐1 (CPT‐1) and carnitine acyl carnitine transposition enzyme in the liver and adipose tissue, which are all involved in lipid metabolism of decomposition of the enzyme.^131^, ^132^ RES inhibits nutritional reprogramming of liver proteome in mice and hinges on the down‐regulation of mitochondrial effects on carbohydrates and fats. Dietary components have prodigious effects on the liver proteome, not only on metabolic pathways, but also on mitochondrial function and RNA splicing.^133^ DMY has been reported to protect HUVEC and HepG2 cells from oxidative stress injury by altering the mitochondrial apoptosis pathway involving Bcl‐2, Bak and caspase‐9/caspase‐3, while stimulating autophagy and reducing lipid accumulation and lipogenesis in vitro.^134^ DMY also mends NAFLD by inhibiting hepatic mitochondrial dysfunction and REDOX homeostasis, and the underlying mechanism may be the up‐regulation of SIRT3 expression due to the activation of AMPK‐peroxisome proliferator‐activated receptor‐γ coactivator‐1 alpha (PGC1α)/oestrogen‐related receptor‐α (ERRα) signalling pathway. What is more, studies have provided a new finding that apart from regulating SIRT3, the activation of SIRT1 also participates in the DMY treatment of fatty liver disease. Its elemental mechanisms involve the regulation of key regulators of lipid metabolism, oxidative stress, inflammation and fibrosis. These beneficial effects may be caused by the activation of the SIRT1‐mediated signalling cascade in the liver.^135^, ^136^ These results suggest that DMY lays the groundwork for new prevention and treatment strategies for NAFLD.

### 3.5 Polyphenols affect metabolism through liver inflammation

Inflammation is a defensive adaptive response of the host to infection, cellular stress or tissue damage, regulated by multiple signalling pathways to ensure the initiation, maintenance and resolution of the inflammatory process. When inflammatory response is unbalanced in the body, the excessive inflammatory response can impair healthy tissues and cells, and even lead to the loss of normal organ function in severe cases.^137^ Substances that are naturally present in food and plants, including polyphenols, play a biological role in regulating inflammatory processes. In general, CGA can meliorate liver function, reduce liver tissue pathological damage, hepatic inflammatory response and liver fibrosis, and then affect liver cancer. These effects may be mainly through the inhibition of toll‐like receptor 4 (TLR4) signal activation, adhesion molecule expression, liver leukocyte infiltration and activation, as well as the production of pro‐inflammatory cytokines.^138^ In addition, CGA indirectly influences the development of liver cancer by inhibiting the activation of NF‐κB and JNK/AP1 and blocking the signalling pathway of transforming growth factor‐β1 (TGF‐β1)/SMAD Family Member 7 (SMAD7) regulated by miR‐21.^139^, ^140^ Supplementation of DMY may recuperate glucose and lipid metabolism, along with various biochemical indicators in patients with NAFLD, and the therapeutic effects of DMY treatment may be the result of increased insulin resistance and decreased serum levels of TNF‐α.^141^ EGCG precludes the development of experimental NASH induced by a high‐fat diet, which seems to exert lipid metabolism and anti‐oxidant properties. EGCG supplementation also trims steatosis, inflammation, CYP2E1 and alpha‐smooth muscle actin (α‐SMA) levels.^142^ RES can minify fat production by inhibiting SREBP‐1c and FAS or increasing the activity of anti‐oxidant enzymes, thereby treating NAFLD.^131^, ^132^ In chronic HBV infection, mitochondrial function and proteostasis are dysregulated in depleted HBV‐specific CD8+ T cells with impaired phagocytosis. RES and oleuropein (OLE) in CD8+ T cells compel improvements in mitochondrial, protein homeostasis and anti‐viral function, including metabolites produced by RES and OLE metabolism in vivo, may even further improve functional T cells recovery by mitochondrial‐targeted antioxidants.^143^ CUR can block the PI3K/AKT pathway to inhibit EMT and angiogenesis. CUR also activates innate immune cells, inhibits EMT by regulating IL‐6/JAK/STAT3 and IL‐1β/NF‐κB pathways, hampers anaerobic glycolysis by inhibiting lactate dehydrogenase (LDH) and Hypoxia inducible factor 1‐alpha (HIF‐1α), and reduces lipid synthesis by down‐regulating FASN and up‐regulating serum the high‐density lipoprotein cholesterol (HDL‐C) and mRNA levels of apolipoprotein A1 in the sorafenib‐treated mice. Collectively, CUR enhances the anti‐tumour efficacy of sorafenib by activating immune function, down‐regulating EMT and reversing metabolic derangements.^144^, ^145^

Besides its efficient anti‐inflammatory and anti‐oxidant effects, QUE also has a better protective effect on NAFLD by improving abnormal lipid metabolism. Studies have shown that QUE significantly down‐regulates TXNIP, bringing about the inhibition of NLRP3 inflammasome activation, augment of PPARα and suppression of SREBP‐1c, SREBP‐2, FAS and LXRα, and decrease of ROS and interleukin‐1β in the liver of diabetic rats. This inhibitory effect on liver TXNIP helps to improve liver inflammation and lipid accumulation under hyperglycemic conditions, and can prevent NAFLD related to type 1 diabetes.^146^ Another protective mechanism of QUE on NAFLD is through the regulation of intestinal flora imbalance, related intestinal‐liver axis activation and lipotoxic blockade, as well as the subsequent resistance to inflammasome response and the mediation of activating the reticular stress pathway. QUE supplements reduce lipid accumulation in the liver by regulating lipid metabolism gene expression, CYP2E1‐dependent lipid peroxidation and related lipotoxicity, thereby suppressing insulin resistance and NAFLD activity scores. It reverses the intestinal flora imbalance and related endotoxemia‐mediated TLR4‐NF‐κB pathway, and subsequently represses the inflammasome response and the activation of the reticulum stress pathway, leading to lipid obstruction.^147^ The regulatory effect of QUE is at least partially mediated through the inactivation of the PI3K/AKT pathway, conducing to muffle lipid accumulation by inhibiting the transport of fatty‐acid translocase (FAT)/cluster of differentiation 36 (CD36) to the plasma membrane. Moreover, it can induce the expression of small heterodimer partner (SHP) in vivo, an orphan nuclear receptor, related to the increase of neutral lipid accumulation that characterizes the steatosis state.^148^ Gluconeogenic enzyme fructose 1,6‐bisphosphatase 1 (FBP1) loss disrupts hepatic metabolism and promotes HCC development through the hepatic stellate cell senescent secretome, and treatment with dasatinib/quercetin can target the elimination of senescent HSCs driven by hepatic FBP1 loss, dampening tumour progression.^149^ Polyphenols directly participate in the regulation of inflammatory responses. As effective antioxidants, they can remove free radicals and squelch their formation, thereby indirectly exerting anti‐inflammatory effects. In general, the anti‐inflammatory properties of polyphenols and their metabolically active substances have become a research hotspot in recent years, and can play a role in a variety of cells and sites through different mechanisms.

### 3.6 Polyphenols affect metabolism through toxic metabolites

**FIGURE 5:**
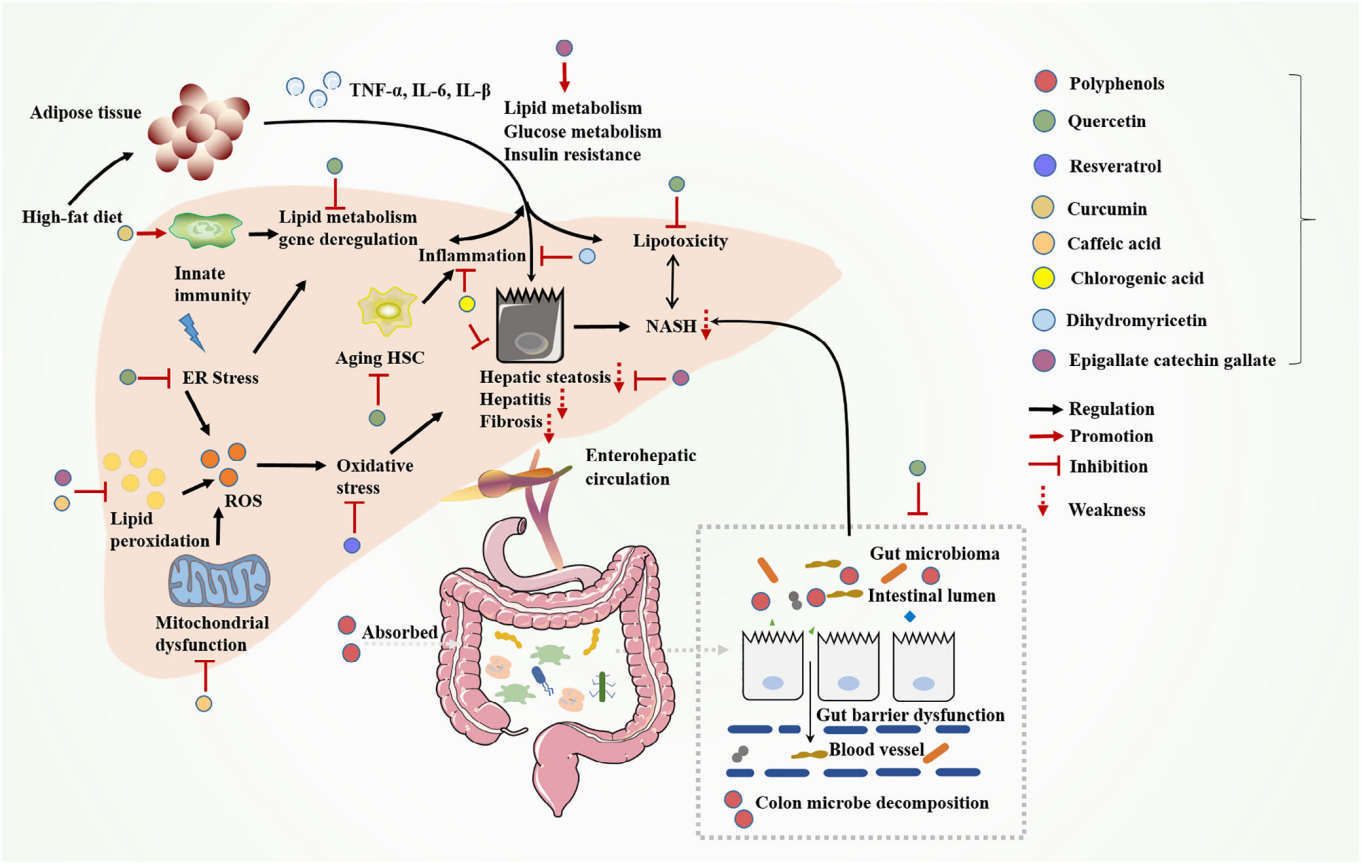
Polyphenols intervene in the progression of liver disease and NASH in miscellaneous pathways. Most polyphenols are metabolized in the liver, at the same time microorganisms in the colon metabolize polyphenols into small molecules that enter the liver. Polyphenols directly or indirectly interfere with metabolism by affecting oxidative stress, inflammation, immunity and liver‐enteric circulation.

The liver is an extraordinary metabolic physiological organ; hence, liver damage causes serious harm to the body and severe liver dysfunction, even causes liver cirrhosis and liver cancer.^150^ The liver protects the body through its own phagocytosis and detoxification, and natural phenolic compounds exert hepatoprotective effects to treat obstructive jaundice liver injury, acute liver injury, alcoholic liver injury and liver cancer.^151^ The strong anti‐oxidant capacity of polyphenols is generally attributed to the hydroxyl radicals present in their structures, which can well quench free radicals, integrate metal ions, induce anti‐oxidant enzyme activity, and modulate anti‐oxidant cell signalling pathways in vivo, and can also effectively prevent the body damage caused by heavy metals.^152^, ^153^ In miscellaneous models, CGA has been proven to be valuable in protecting many liver diseases, such as CCl_4_‐induced liver fibrosis and acute liver injury, presumably via boosting Nrf2‐mediated anti‐oxidant pathway and blocking NLRP3 inflammasome activation, and cetaminophen‐induced and d‐galactose‐induced liver injury.^154^ The researchers found that CGA reduced histopathological lesions and markers of liver injury, such as alanine aminotransferase, aspartate aminotransferase, alkaline phosphatase, total bile acid, total cholesterol, high‐density lipoprotein cholesterol and low‐density lipoprotein cholesterol.^155^ It has been shown that the protective effect of DHM on APAP‐induced hepatotoxicity has multi‐target and multi‐pathway characteristics involving APAP metabolism, lipid regulation, along with hepatocyte death and regeneration. DHM relieves APAP‐induced liver injury in mice by inhibiting hepatocyte death, promoting p53‐related protein regeneration, regulating PPARs and SREBP‐1c mediated lipid homeostasis and APAP transformation.^156^ QUE has a significant inhibitory effect on drug metabolizing enzymes. It can withhold the formation of N‐acetyl‐p‐benzoquinone imine (NAPQI), which is a toxic metabolite of acetaminophen in rats and isolated rat liver cells, thereby preventing CYP2E1 and other CYPs mediated inhibition of paracetamol metabolism, and reducing the oxidative stress caused by paracetamol in the liver and kidneys.^64^ Ethanol incubation of human primary hepatocytes results in sustained glutathion depletion, lipid peroxidation and membrane damage, whereas QUE‐induced HO‐1 correspondingly reduces ethanol‐derived oxidative damage. HO‐1 induction intercedes the protective effect of QUE via p38, especially via ERK/Nrf2 transduction pathway in human hepatocytes.^157^ CUR, with anti‐inflammatory activity, also exhibits anti‐cancer activity through a variety of mechanisms, including inhibiting the initiation, progression, invasion and metastasis of tumour cells. CUR can weaken liver cancer induced by diethylnitrosamine through intervening oxidative response kinases, inflammatory factors and reducing metabolic disorders. It also dwindles liver toxicity in rats caused by galactosamine or CCl_4_, and prevents the carcinogenic effects of liver carcinogens aflatoxin or nitrosodiethylamine, relieving liver fibrosis in rat models of NASH.^158^ Iron chelator has been shown to form redox active iron complexes, with iron depleted, sequentially exerting anti‐tumour effects. Studies have found that CUR is an efficacious inhibitor of iron toxicity in T51B cells, incarnating in reducing iron‐dependent oxidative stress and iron toxicity in T51B rat liver epithelial cells without preventing iron uptake. It deters iron‐induced ROS production and iron signalling to multiple oxidative stress response conductions. There is evidence that hepatocytes treated with CUR exhibit the characteristics of iron consumption, which affects the iron metabolism protein in cultured normal hepatocytes and attenuates the level of ferritin in the liver.^124^, ^159^, ^160^ EGCG mitigates the deterioration of liver injury in the model of NAFLD associated with lower concentration of pro‐fibrogenic, oxidative stress and pro‐inflammatory mediators partially through counteracting the activities of TGF/SMAD, PI3K/AKT/FoxO1 and NF‐κB pathways. Therefore, polyphenols are useful supplements in the prevention of NAFLD and liver injury (Figure 5).^161^

## 4 CONCLUSIONS AND FUTURE PERSPECTIVES

It is well known that the occurrence and development of liver cancer is a multi‐gene, multi‐step and multi‐stage process.^162^ New phenomena and mechanisms have been discovered to generate some new tumour prevention and control strategies for metabolic regulation. Metabolic changes provide selective advantages for tumour growth, proliferation and survival, such as increasing energy production, macromolecular biosynthesis and maintaining redox balance.^163^ The regulation of metabolic reprogramming in cancer is eminently convoluted and may occur through genetic mutation and epigenetic regulation.^164^ The liver is the most imperative energy metabolism organ in the human body. A bounty of liver‐related disease is directly related to disorders of liver energy metabolism through distorting inflammation and fibrosis.^165^ The transition from oxidative phosphorylation to the glycolytic pathway in hepatocarcinoma fulfils the need for expeditious cell proliferation and provides a sustained microenvironment for tumour development. The liver plays a key role in lipid metabolism and maintenance of plasma lipoprotein homeostasis through lipid sensing regulators and lipid regulating enzymes.

Increased initio synthesis of fatty acids in tumour cells is a significant feature of the deterioration of cancer, substantially due to the up‐regulation of multiple levels of lipid‐related genes in transcription, translation and post‐translational modification, enzyme activity, as well as the influence of changes in these genes or lipid metabolism on the expression of oncogenes. The momentous genes FAS, ACC, ACLY and the crucial transcriptional regulatory factor SREBP1 that adjust their expression and activity can be used to inhibit tumour genesis, and the growth of tumour cells can be effectively reduced by corresponding gene knockout or chemical inhibitors.^166^, ^167^ Glucose metabolism is one of the basic biochemical processes in mammals, specifically regulated by tissues and cells, involving many transporters, enzymes and transcription factors. Subsidence of glucose flux by key enzymes such as LDH and 6‐phosphofructose 2‐kinase can effectively decrease the incidence of tumours.^168^ Studies have shown that high‐fat diet, diabetes and obesity are related to the occurrence and development of NAFLD‐related hepatocellular carcinoma. These metabolic factors are closely related to insulin resistance and hyperinsulinemia, inducing cell proliferation and inhibiting apoptosis through PI3K and MAPK pathways, and playing an important role in the carcinogenesis of HCC.^169^, ^170^

Regulation of fatty acids is critical in the development of NAFLD and liver cancer. In the regulation of lipid metabolism by polyphenols, such as DMY, RES, CA and catechins, AMPK plays a key role in cellular energy sensors. Activated AMPK, on the one hand, affects the protein expression level of FAS to adjust fatty acid synthesis, mainly by down‐regulating SREBP‐1c. On the other hand, it increases the phosphorylation level of ACC to attenuate activity, thereby reducing its ability to participate in fatty acid production, and then up‐regulates the expression level of the fatty acid oxidation rate‐limiting enzyme CPT‐1. It is also closely related to fat oxidation, promoting fatty acid oxidation. Bounteous polyphenols such as DMY, RES, ECG, EGCG and QUE can directly regulate SREBP. SREBP is mainly involved in fatty acid synthesis and glucose metabolism, lipogenic transcription factors regulated by insulin and glucose, responsible for regulating key genes involved in hepatic lipogenesis, such as FAS, ACC, LDL receptors, etc. PPARs also play a key role in the regulation of metabolism of polyphenols. PPARα directly binds to polyunsaturated fatty acids and enhances the activity of fatty acyl‐CoA oxidase, thereby promoting mitochondrial oxidation of fatty acids. Insulin resistance is a common feature in NAFLD patients and is also pivotal to its pathogenesis. Polyphenols can significantly improve insulin resistance, which is manifested by decreased glucose deposition in non‐liver tissues. Insulin resistance in adipose tissue leads to dysregulated lipolysis, causing inappropriate release of fatty acids, which in turn impresses impaired insulin signalling throughout the body. It can be seen that a variety of polyphenolic compounds can regulate key transcription factors and signalling pathways in the body's metabolism, and play an important role in the treatment of NAFLD and liver cancer. Overall, in liver cancer cells, polyphenols inhibit proliferation and migration through promoting SIRT1‐mediated post‐translational modification of the PI3K/AKT pathway; and the AMPK‐Lipin1 signalling pathway is involved in the protection of cells; and polyphenols activate the AMPK/PPARα signalling pathway to hinder the synthesis of TG and increase the oxidation of fatty acids in the hepatic cell steatosis model, thereby weakening liver steatosis.

Polyphenols have various biological regulatory effects on metabolism and involve a variety of potential regulatory mechanisms, so they can be used in the prevention and treatment of oxidative stress, inflammation, aging and other pathophysiological processes of liver diseases, demonstrating protective effect. The idiographic molecular mechanism of the protective effects of diversified polyphenols on the liver is by affecting lipid metabolism, glucose metabolism, mitochondrial metabolism, oxidative stress, toxic metabolites, liver inflammation and other comprehensive metabolic reactions to inhibit liver cancer, involving multiple representative polyphenols, such as chlorogenic acid, caffeic acid, dihydromyricetin, quercetin, catechins, curcumin and resveratrol. Currently, an increasing number of polyphenols have been used as anti‐cancer agents to prevent and treat liver diseases such as NAFLD, drug‐induced liver injury, liver cancer and alcoholic liver disease. RES, EGCG or CUR are more widely used, and any one of these compounds may provide healthy properties to the liver.^171^ Preclinical and clinical settings have shown many roles of polyphenols, such as increased fatty acid oxidation as well as insulin resistance, modulation of glucose metabolism, anti‐oxidation and remission of inflammation, which represent the major pathogenic steps from the onset of NAFLD to liver cancer.^172^, ^173^ In addition, polyphenols as dietary supplements may provide more straightforward adherence strategies in the prevention or amelioration of ameliorating chronic and acute liver diseases. As natural antioxidants, polyphenols exhibit favourable anticancer activity in many aspects and have basically no toxic and side effects on the human body.^174^ Despite their considerable therapeutic potential, the poor stability and low bioavailability have hindered their application in vivo. The modification of polyphenols or the application of nano‐formulations, the synergistic effect of polyphenols with drugs or multiple chemotherapeutic drugs, and targeted therapies can confer great advantages in terms of a better bioavailability of polyphenol components and achieve better therapeutic effects.^175^, ^176^ However, at present, data from clinical studies are still limited. There are many types of polyphenols, and their systemic mechanisms and related effects in regulating the response pathways of various organisms to external noxious stimuli are still insufficient. How to improve the bioavailability of polyphenols, as well as many questions concerning the use of polyphenols in the clinical setting remain unanswered. Therefore, it is worthwhile for researchers to continue to explore and evaluate how the biological protection of polyphenols with therapeutic potential and a wide range of natural sources can exert their clinical application value more effectively and safely.

In conclusion, polyphenols protect the liver and inhibit the development of liver cancer by affecting metabolism, which provides a scientific basis for the use of polyphenols in clinical and dietary medicine.

## AUTHOR CONTRIBUTIONS

Haiyang Yu and Tao Wang conceptualized the review. Shuangfeng Li and Shuangshuang Yin read relevant literature and wrote the article. Hui Ding, Yingying Shao, Shiyue Zhou, Weiling Pu and Lifeng Han critically reviewed and edited the manuscript. All authors have read and approved the final manuscript.

## CONFLICT OF INTEREST

The authors declare no conflicts of interest.

## Data Availability

Data sharing is not applicable to this article as no new data were created or analyzed in this study.
